# IH-TCGAN: Time-Series Conditional Generative Adversarial Network with Improved Hausdorff Distance for Synthesizing Intention Recognition Data

**DOI:** 10.3390/e25050781

**Published:** 2023-05-11

**Authors:** Siyuan Wang, Gang Wang, Qiang Fu, Yafei Song, Jiayi Liu

**Affiliations:** Air Defense and Antimissile School, Air Force Engineering University, Xi’an 710051, China

**Keywords:** intention recognition, multivariate time series, data augmentation, generative adversarial network, Hausdorff distance

## Abstract

As military technology continues to evolve and the amount of situational information available on the battlefield continues to increase, data-driven deep learning methods are becoming the primary method for air target intention recognition. Deep learning is based on a large amount of high quality data; however, in the field of intention recognition, it often faces key problems such as low data volume and unbalanced datasets due to insufficient real-world scenarios. To address these problems, we propose a new method called time-series conditional generative adversarial network with improved Hausdorff distance (IH-TCGAN). The innovation of the method is mainly reflected in three aspects: (1) Use of a transverter to map real and synthetic data into the same manifold so that they have the same intrinsic dimension; (2) Addition of a restorer and a classifier in the network structure to ensure that the model can generate high-quality multiclass temporal data; (3) An improved Hausdorff distance is proposed that can measure the time order differences between multivariate time-series data and make the generated results more reasonable. We conduct experiments using two time-series datasets, evaluate the results using various performance metrics, and visualize the results using visualization techniques. The experimental results show that IH-TCGAN is able to generate synthetic data similar to the real data and has significant advantages in the generation of time series data.

## 1. Introduction

Target intention recognition is a core component of situational cognition. Target intention recognition is essentially a pattern recognition problem in dynamic, adversarial conditions. Intention recognition requires a series of highly abstract and complex thinking activities, such as key feature extraction, comparative analysis, association, and reasoning, to achieve accurate target intention recognition based on professional knowledge and relevant experience, considering key information such as the battlefield environment, target attributes, and target status. Intention recognition often faces critical problems, such as low data volume and unbalanced datasets due to the lack of actual combat situations.

One possible solution to the above problem is to generate more synthetic data based on existing real data. The intention recognition data are multivariate time-series data, so we then need to solve the multiclass, multivariate time-series generation problem. To address this problem, many experts and scholars have utilized the generative adversarial network (GAN). GAN was first proposed by Goodfellow [1] in 2014. GAN is a network structure with an adversarial idea, containing a generator and a discriminator that confront each other. The role of the discriminator is to try to distinguish between real data and synthetic data, and the role of the generator is to try to improve itself so as to generate data that can confuse the discriminator. When the discriminator is unable to distinguish between true and false data, the generator is considered to have achieved a good generation result at this point. For the problem of temporal data generation, many kinds of GAN variants have been proposed in recent years, such as TimeGAN [2,3], MTSS-GAN [4], RGAN [5], RCGAN [5], PART-GAN [6], PSA-GAN [7], CEGEN [8], etc. In addition, MAD-GAN [9], FGANomaly [10], DoppelGANger (DG) [11], etc., have been proposed for the anomaly detection of temporal data. For other applications of temporal data, the TimeVAE synthesis method, similar to TimeGAN [12], CGAN-based temporal simulation [13], the synthetic multivariate time-series generation method for flare forecasting [14], and time series forecasting for hourly photovoltaic power using CGAN and Bi-LSTM [15], is proposed. However, most of these GAN variants are suitable for time-series data correlation problems without class conditions or contain only positive and negative class conditions, which are not applicable to the multiclass, multivariate time-series generation problems we have mentioned.

To generate multivariate time-series data with multiple intentions, we propose a new IH-TCGAN method in this paper. Specifically, we first propose a time-series conditional generative adversarial network (TCGAN). This improves the discriminator in the basic GAN into a transverter which can transform real data and synthetic data into the same manifold for comparison. This prevents the transformation process from being difficult because of different intrinsic dimensions. Second, we add a restorer and classifier following the transverter. The transverter can represent the underlying temporal features in the data by lower dimensional features, while the restorer can ensure that this transformation process is reversible. The classifier can distinguish the data from different classes, which prompts the model to eventually generate synthetic data under multiple intention labels. The loss functions of GAN and its variants generally pay little attention to the differences in the time order of the temporal data, which leads to a large deviation in the final, generated temporal data. To solve this problem, we then proposed an improved Hausdorff distance as the loss function of TCGAN. We call the TCGAN that introduces the improved Hausdorff distance the IH-TCGAN. This is achieved by adding a time regularization term to the ordinary Hausdorff distance formula, which can characterize the difference in time order. The improved Hausdorff distance enables a more accurate measurement of the distance between two temporal sample sets, prompting IH-TCGAN to generate higher quality synthetic samples. Finally, we perform experimental validation using the human activity recognition dataset and the target intention recognition dataset. We evaluated the experimental results using multiple metrics and performed visualization.

The main contributions of this paper are summarized as follows:

1. We propose a variant of GAN for generating multiclass, multivariate time-series data called TCGAN. We improve the discriminator into a transformer and add a restorer and a classifier to the network structure. TCGAN provides new ideas and methods for solving multivariate time-series data generation problems under multiclass conditions.
2. We propose an improved Hausdorff distance as the loss function of TCGAN. The improved Hausdorff distance can better represent the temporal similarity between real and synthetic data, which prompts IH-TCGAN to generate higher quality samples. The improved Hausdorff distance can also be used in other domains where temporal data discrepancy metrics are required.
3. We perform experimental validation on multiple time-series datasets. Compared with other methods, IH-TCGAN is able to generate more realistic and diverse multivariate time-series data. The intention recognition data it generates can be used to train a data-driven intention recognition model with good results.

The rest of the paper is organized as follows. Section 2 briefly describes the air-target intention recognition problem and reviews GAN and its progress in time series. Section 3 presents the general architecture and the functions of each part of our proposed TCGAN. Section 4 introduces the improved Hausdorff distance with a time regularization term. Section 5 is devoted to the experiments and analysis. The conclusion is in Section 6.

## 2. Related Works

### 2.1. Description of Target Intention Recognition

Air target intention recognition refers to the combination of the analysis of information collected through various sensors in a dynamic, adversarial environment and commander-related knowledge and operational rules to infer the tactical intentions of air targets. The core issue of target intention recognition is to clarify the target’s intention space and the target’s feature information.

Intention space refers to the set of possible intentions of the air targets for different scenario settings. The feature information is the temporal feature variables about the target obtained by sensor acquisition and fusion. In this paper, we refer to the setting in Ref. [16], and the intention recognition dataset is similar to that in Ref. [16]. The intention of the target in the intention recognition dataset is divided into six types: attack, reconnaissance, surveillance, cover, interference, and retreat. There are 12 types of target characteristic information: height, velocity, acceleration, heading angle, azimuth, distance, course shortcut, one-dimensional range profile, radar cross section area, air-to-air radar state, air-to-ground radar state, and electronic interference state. More specific information is provided in Section 5.1.

### 2.2. GAN and Variants

**Figure 1:**
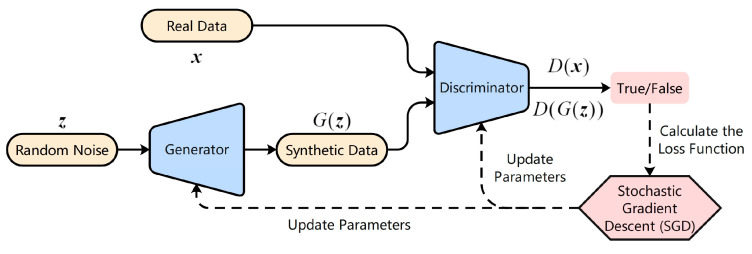
The general architecture of GAN.

GAN was first proposed by Goodfellow [1] in 2014. GAN is an unsupervised generative model with adversarial ideas. GAN consists of two parts, a discriminator (D) and a generator (G), which can be various nonlinear mapping functions, such as machine learning models and deep neural networks. The role of the discriminator is to try to distinguish between real data and synthetic data, and the role of the generator is to try to improve itself so as to generate data that can confuse the discriminator. During the training process, the discriminator and the generator evolve alternately until the two reach Nash equilibrium. At this point, the discriminator can no longer distinguish the true data and the false data, indicating that the generator can generate data similar to the true data and the generator has achieved a good generation effect. The general architecture of GAN is shown in Figure 1.

The basic idea of GAN is a min–max game between the generator and the discriminator. The loss function of the basic GAN is as follows:

(1) $$\underset{G}{\min}\;\underset{D}{\max}\;L(D,G)=E_{x\sim p_{r}(x)}\left[\log D(x)\right]+E_{z\sim p_{z}(z)}\left[\log(1-D(G(z)))\right]$$

where x represents the real sample and $p_{r}(x)$ is the data distribution of the real sample. z represents the random noise and $p_{z}(z)$ is the data distribution of the noise. G(z) represents the sample generated by G and E represents the expectation.

**Figure 2:**
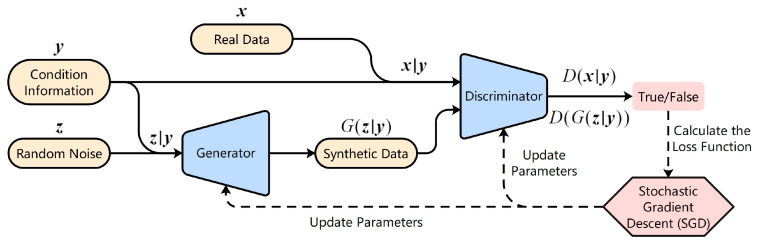
The general architecture of CGAN.

Conditional Generative Adversarial Network (CGAN) [17] is a variant of GAN with conditional constraints. This network is structured to introduce conditional variables in the generator and discriminator, separately, which can effectively guide the training process of the generator. The conditional variables here can be any type of information that helps to capture the features of the real data distribution, such as category labels in image recognition, information about a particular feature, or data from other modalities [18]. The loss function of CGAN is as follows:

(2) $$\underset{G}{\min}\;\underset{D}{\max}\;L(D,G)=E_{x\sim p_{r}(x)}\left[\log D(x|y)\right]+E_{z\sim p_{z}(z)}\left[\log(1-D(G(z|y)))\right]$$

where (x|y) is the real sample with condition y and (z|y) is the random noise with condition y. CGAN makes GAN change from an unsupervised network to a supervised network, which can solve the problem of data generation with specific labels. The general architecture of CGAN is shown in Figure 2. Since our goal is to generate time series data under multiple intention labels, CGAN, which can control the pattern of generated samples, becomes the basic framework used in our study.

To solve the instability and mode collapse problems during GAN training, Yu Xue et al. [19,20,21] proposed PEGAN with a self-attentive module to improve on the disadvantages of convolutional operations. During the training process, the discriminator will play against multiple generators simultaneously, where each generator adopts a different objective function as a mutation operation. Every time after the specified number of training iterations, the generator individuals will be evaluated and the best performing generator offspring will be retained for the next round of evolution. Based on this, the generator can continuously adjust the training strategy during training, and the self-attention module also enables the model to obtain the modeling ability of long-range dependencies.

In response to the temporal characteristics of the input data, many experts and scholars have proposed many new methods for time-series generation problems based on GAN and CGAN. Derek Snow [4] proposed a new generative adversarial network, MTSS-GAN, designed to simulate different multivariate time-series data. MTSS-GAN consists of stacked GANs with simplified processing. Stephanie L. Hyland et al. [5] proposed a recurrent GAN (RGAN) and recurrent conditional GAN (RCGAN) for real-valued (medical) time-series generation to generate a real multidimensional time series. RGAN and RCGAN use recurrent neural networks in the generator and discriminator. Shuo Wang et al. [6] proposed a practical privacy-preserving generative model, PART-GAN, which can be used for time-series data expansion and sharing. Paul Jeha et al. [7] proposed a progressive self-attention GAN (PSA-GAN), which uses progressively growing GAN and self-attention to generate high-quality, long time sequence samples.

**Figure 3:**
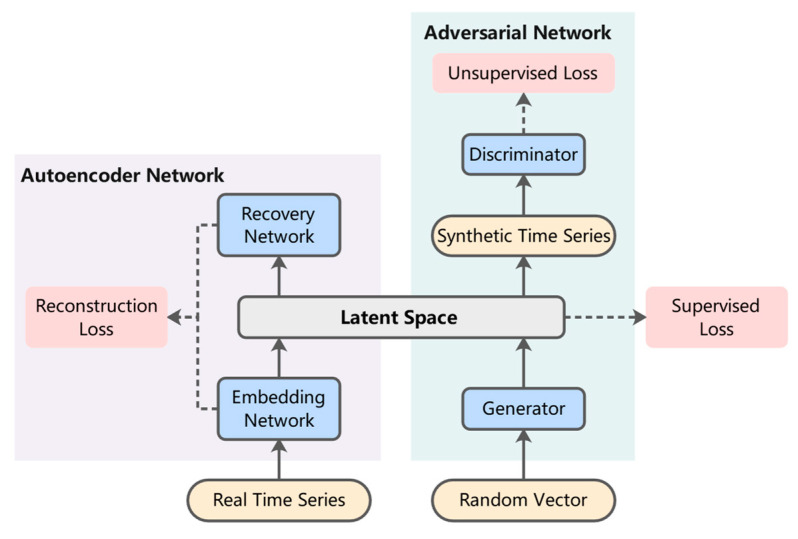
The general architecture of TimeGAN.

In 2019, Yoon proposed a new framework for generating realistic time-series data, called TimeGAN [2]. TimeGAN adds an embedding network and a recovery network to the normal GAN, combining the flexibility of unsupervised mode with the control provided by supervised training. The general architecture of TimeGAN is shown in Figure 3.

In addition to the unsupervised loss between true and false samples in the basic GAN network, TimeGAN incorporates supervised loss using the original data as a supervised term, which allows the model to capture the conditional distribution in the sequence data. The autoencoder network can provide reversible mapping between features and potential representations, reducing the high dimensionality of the adversarial learning space. By jointly training the embedding network and the generator network, the supervised loss can be minimized in order to make the latent space not only improve the parameter efficiency, but also facilitate the generator to learn the temporal relationship by specific conditions. TimeGAN proposes an idea to compare real and synthetic data in a low-dimensional latent space. In addition, the autoencoder network in TimeGAN provides a guarantee for the correct mapping of real data to potential space.

After analyzing the above methods, we think that the design of the autoencoder network and latent space in TimeGAN has great advantages. The purpose of GAN is to make the distribution of synthetic data as similar as possible to the distribution of real data. However, there is a more serious problem with TimeGAN. The dimensions of the real time series after the dimensionality reduction by the embedding network and the synthetic time series generated by the generator may not be the same. The dimensions of the real time series after the dimensionality reduction by the embedding network and the synthetic time series generated by the generator may not be the same. The two are not in the same dimensional manifold, which will lead to difficulties in fitting the distributions of the synthetic data and the real data. In addition, TimeGAN is unable to generate multiclass time-series data in the presence of labels. To solve the problem of fitting difficulties between different time-series distributions and the generation of multiclass intention recognition data, we propose a new multiclass, multivariate time-series data generation network, IH-TCGAN. Improvements in the network structure are described first, followed by improvements in the loss function.

## 3. Time-Series Conditional Generation Adversarial Network

### 3.1. General Architecture

**Figure 4:**
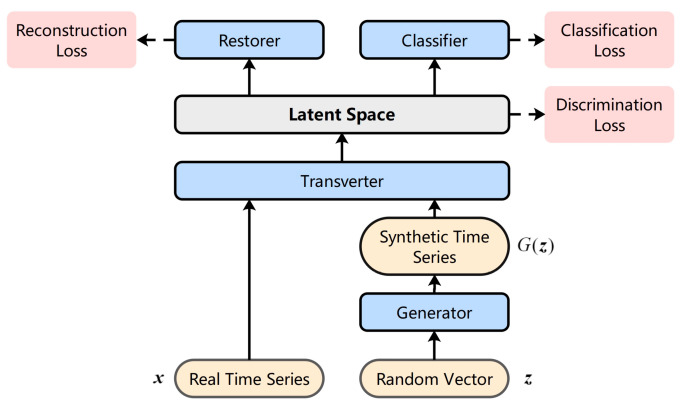
The general architecture of TCGAN.

In order to be able to generate high-quality multiclass intention recognition temporal data, we propose a variant of GAN, called TCGAN. TCGAN consists of four networks: generator, transverter, restorer, and classifier. It has three loss functions: discrimination loss, reconstruction loss, and classification loss. The general architecture of TCGAN is shown in Figure 4.

In the following, we describe each part of TCGAN in detail. First, we provide an introduction to the transverter. The concept of the manifold of the data is elaborated, which is the main reason why we designed the transverter. Later, a specific description of the restorer and classifier is given. Finally, we introduce the joint training mechanism of TCGAN.

### 3.2. Transverter and Discrimination Loss

The data of a dataset often lie on different manifolds. A manifold is a space with local Euclidean space properties [22,23]. We can use neural networks for manifold learning. The view of manifold learning assumes that the data that can be observed are actually mapped from a low-dimensional manifold to a higher-dimensional space. Due to the limitation of the internal features of the data, some data in high dimensions will generate dimensional redundancy and actually need only a relatively low dimension to be uniquely represented. Borrowing from this idea, we generate the intention recognition data based on GAN.

Intention recognition data are multivariate time-series data, which are often in a high-dimensional manifold space. We assume that the real data are x and the synthetic data are G(z). z denotes the noise of the generator input. $p_{r}$ and $p_{G}$ are the distributions of x and G(z), respectively, and $p_{z}$ is the distribution of the noise z. The purpose of the generator is to generate G(z) that is as similar to x as possible, i.e., to make the distribution $p_{G}$ as similar to $p_{r}$ as possible. In this way, the discriminator is tricked so that it cannot distinguish the real and synthetic data. A dataset that lies in a high-dimensional manifold can often be projected into a low-dimensional manifold. The smallest dimension in this low-dimensional manifold is the intrinsic dimension [24]. If the real data and the synthetic data have different intrinsic dimensions, then the manifolds of the two must be different, and it is difficult to convert $p_{G}$ to $p_{r}$.

We assume that the intrinsic dimensions of the manifolds of real data x and synthetic data G(z) are $n_{r}$ and $n_{G}$, respectively. The noise $z=\{z_{1},z_{2},\ldots ,z_{m}\}$ of the generator input obeys a simple noise distribution $p_{z}$, where m is the dimensionality of the noise. The purpose of the generator is to convert z to x as much as possible. However, the noise z is usually just a low-dimensional simple noise (e.g., each item in $z=\{z_{1},z_{2},\ldots ,z_{k}\}$ may be linearly correlated) with dimension m smaller than that of the real data x, while the intrinsic dimension $m^{\prime }$ of the noise may even be smaller than m. We input z into the generator G to obtain the synthetic data G(z). The intrinsic dimension $n_{G}$ of the manifold that it is in will be constrained by $m^{\prime }$, and $n_{G}$ can only be smaller than the intrinsic dimension $n_{r}$ of the real data x. In this case, smaller intrinsic dimensions cannot carry more data features, and forcing the construction of a manifold with higher intrinsic dimensions will result in the loss of data information. The purpose of GAN is to simulate the distribution situation, and the difference in the intrinsic dimensions of the two makes the process of transformation from distribution $p_{G}$ to distribution $p_{r}$ difficult.

Based on the above analysis, we consider first transforming the real data x and the synthetic data G(z) into a space with the same intrinsic dimension, i.e., first transforming them into the same manifold. In this way, comparing the two in this manifold space with the same intrinsic dimension will enable a better transformation of $p_{G}$ into $p_{r}$. To construct such a manifold space with the same intrinsic dimension, we design a transverter *T* based on the basic GAN. The transverter T can downscale the temporal information in a high-dimensional space so that it lies in a low-dimensional manifold space. We do this for three main reasons.

1. The information in the high-dimensional space has redundancy, while the information in the low-dimensional manifold space has no redundancy. Data cannot be spread over the entire high-dimensional space because of its inherent characteristics. For example, if data can only appear on one sphere in three-dimensional space, there will never be data points in the space beyond this sphere. In this three-dimensional space, we need three coordinates to represent the points on the sphere; meanwhile, in a different way, for the sphere we only need to represent it with two parameters: longitude and latitude. Therefore, it can be said that this sphere is an unfolding of a two-dimensional manifold in three-dimensional space. The same is true for time series information in higher dimensions. We can translate intention recognition data in high-dimensional space to low-dimensional manifold space. The reason is that even complex temporal dynamics are often driven by fewer and lower dimensional variables.
2. In a low-dimensional manifold space, the distance between two points can be determined using some simple distance metrics, but not in a higher-dimensional space. For example, we can measure the distance between two cities with a ruler that can be bent on the surface of a globe, but we cannot directly measure a shorter distance between two cities with a straight line across the globe, which is not common sense. The intention recognition data are high-dimensional data. After the dimensionality reduction of the transformer, we can use a simple distance metric to determine the distance between the original data and the synthetic data as a loss function. The distance between the original data and the synthetic data can be reduced by continuous iterative learning, thus achieving the goal of generating high quality samples.
3. The manifold can portray the essential characteristics of the data. A transformer can be seen as a mapping relationship that can downscale intention recognition data from a high-dimensional space to a low-dimensional space without losing key information. Through this mapping, it is possible to input the original data and output the more essential features of the data. This process is similar to compression, i.e., representing as much of the original data as possible with less data. The main feature of deep learning is feature learning. Features are able to represent the intrinsic content of the data. In general, the dimension of the features should be smaller than the data itself, i.e., the intrinsic dimension of the data should be smaller than the dimension of the data.

The nature of the transformer T is a neural network, and as a mapping function it can map real data x and synthetic data G(z) into the same manifold so that all the data have the same intrinsic dimensions. Assume that the transformer T can map the input data ∘ (including x and G(z)) to *h*, where h=δ(wx+b) is the representation in the potential space after data transformation. Since the mapping function T has the same parameters w and b, the potential representations T(x) and T(G(z)) of the real and synthetic data obtained after transformation by T have the same dimensions. According to Ref. [19], the maximum likelihood method is usually used for intrinsic dimension estimation. For neural networks, the manifold of the data is generally learned by minimizing the cross-entropy, i.e., minimizing the cross-entropy is equivalent to the maximum likelihood estimation [25,26]. This shows that the dimension of the data after the neural network T transformation is equal to the intrinsic dimension of the data. Therefore, if T(x) and *T*(*G*(*z*)) obtained after *x* and G(z) pass through the transformer T lie on the same manifold, then they have the same intrinsic dimension. At this point, if it is possible to convert T(G(z)) to T(x) as much as possible, then the distribution $p_{G}$ will also be as similar as possible to the distribution $p_{r}$.

Since the intention recognition data are multivariate time-series data with time-series characteristics, we use an LSTM network as the transverter. It can also be replaced by other mapping functions depending on the case. The transverter *T* actually converts the input high-dimensional data into low-dimensional data, and in this respect its function is similar to that of the encoder part of the autoencoder. The output of the discriminator in a normal GAN is a true/false judgment of the input data. The transverter T can be seen as the result of merging the mapping function, the encoder and the discriminator, where we do not need T to output a true/false judgment of the input data. After the transformation of T, the distance $d^{*}(T(x),T(G(z)))$ between T(x) and T(G(z)) can be used as the discrimination loss function $L_{D}$ of the network, denoted as:

(3) $$L_{D}=d^{*}(T(x),T(G(z)))$$

where T(x) and T(G(z)) denote the real mapping data and the synthetic mapping data transformed into the potential space, respectively. During the training of the network, back propagation is continuously used to update the parameter $\theta_{T}$ of T. As training proceeds, d(T(x),T(G(z))) will converge to 0, at which point T(G(z)) will converge to T(x), i.e., *d*(*T*(*x*), *T*(*G*(*z*))) → 0 ⇒ *T*(*G*(*z*)) → *T*(*x*). When the number of training generations is large enough, the loss function of the network tends to be smooth and we obtain T(G(z)) that is close enough to T(x). At this point, the distribution $p_{G}$ and $p_{r}$ will also be as similar as possible, achieving our goal of generating high-quality intention recognition temporal data.

For the distance $d^{*}$ between two sets we use the improved Hausdorff distance, and the specific improvement method we discuss in Section 4. Up to this point, we have transformed the real data x and the synthetic data G(z) into the same manifold using the transformer *T*, and can continuously optimize our generator using the distance between sets as a loss function. Next, we propose a solution to the problem of whether the transformation process of the transverter T (mapping function T) is reasonable and the multiclass problem of the intention recognition data.

### 3.3. Restorer and Reconstruction Loss

When we transform data that are in a high-dimensional space into a low-dimensional space, some original information must be lost. If the dimensionality of the low-dimensional space is too high, we cannot find the most essential features in the data, and also lose the meaning of dimensionality reduction. If the dimensionality of the low-dimensional space is too low, we can only retain a little information about the data and may lose some important information contained in the original data. The transformer T has the same function as the encoder part in the autoencoder. In order to ensure that the transformation process of the transverter T is reasonable and effective, we refer to the design idea of TimeGAN [2] and add a restorer R after the transverter T. The manifold space in which the data are located after the transverter has been dimensioned down is called the latent space. The role of the transverter is to provide a reversible mapping relationship from the original features of the data to the latent space features, while the role of the restorer is to provide a mapping relationship from the latent space to the original features. The combination of the transverter and the restorer can be regarded as a complete autoencoder, both of which function as an encoder and a decoder, respectively.

The restorer is able to restore the real data T(x) after dimensionality reduction to obtain the restored data R(T(x)). The distance $d^{*}(x,R(T(x)))$ between x and R(T(x)) can be used as the reconstruction loss function $L_{R}$ of the network, denoted as:

(4) $$L_{R}=d^{*}(x,R(T(x)))$$

where x and R(T(x)) are the real data and the recovery data, respectively. The distance $d^{*}$ still uses the improved Hausdorff distance specifically for the time-series data proposed in Section 4. The smaller $L_{R}$ indicates that after the process of dimensionality reduction and recovery, the network successfully learns the essential features in the real data. By reducing the reconstruction loss $L_{R}$, the transformation process of the transverter T (mapping function T) can be ensured to be reasonably efficient. It is noted that the restorer R can also be implemented with an LSTM network. The mapping function of the transformer T and the restorer R can also be replaced with other functions that follow causality, such as RNN, GRU, TCN, etc.

### 3.4. Classifier and Classification Loss

To be able to generate intention recognition data with multiple intention classes, we added a classifier C in the network. The role of the classifier is to classify the latent time-series data T(∘) (both T(x) and T(G(z))) after the transverter has been dimensionally reduced, and to evaluate the similarity among samples with different intention labels. The intention recognition dataset we use is a labeled time-series dataset, so the affiliation probability C(T(∘))=P(C=c|T(∘)) that the current data belong to the intention label c corresponding to that entry can be obtained by the classifier. P denotes the probability. The value of C(T(∘)) is larger when the data input to the classifier is a better match to the corresponding intention label. The value of C(T(∘)) is smaller when the data input to the classifier does not belong to the corresponding intention label. We construct a loss function of the classifier based on this probability, called the classification loss function $L_{C}$. Since we always want to obtain a larger value of the probability C(T(∘)), the log-likelihood of designing the classification loss $L_{C}$ as the affiliation probability C(T(∘)) is taken as negative, denoted as:

(5) $$L_{C}=-E\left[\log C(T(\circ ))\right]=-E\left[\log P(C=c|T(\circ ))\right]$$

where T(∘) denotes T(x) and T(G(z)); c is its corresponding intention label; and E denotes expectation. For classifier C, a mapping function that follows the causal relationship can still be chosen to implement.

### 3.5. Joint Training Mechanism

**Figure 5:**
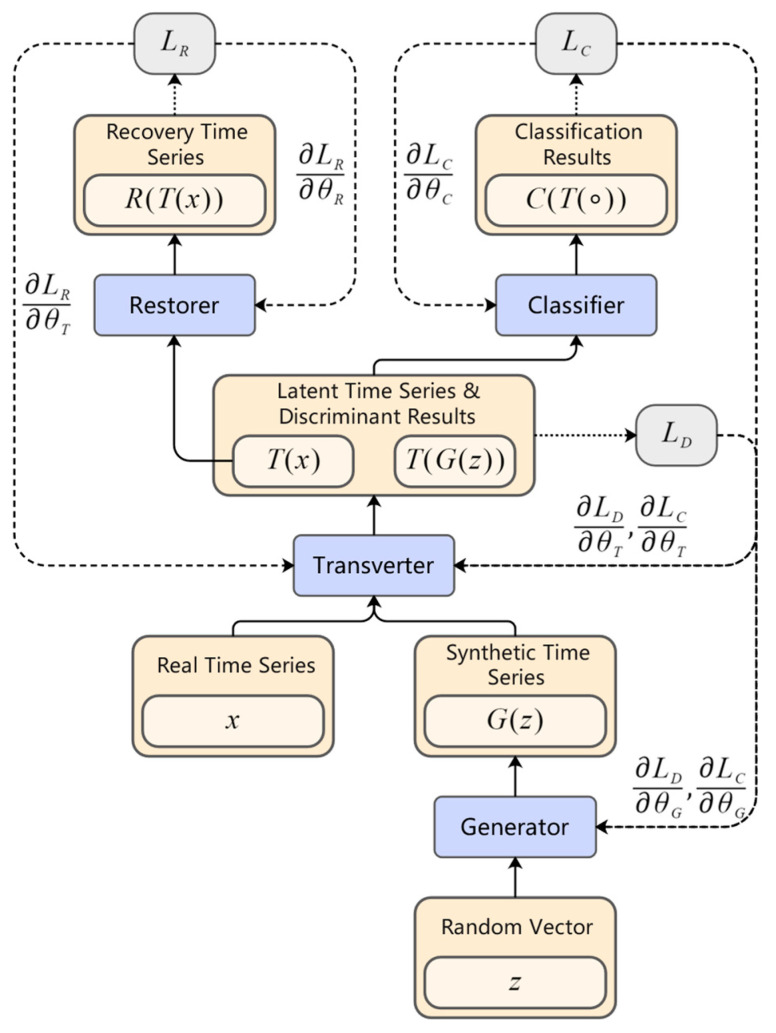
Training mechanism of TCGAN.

TCGAN has three types of loss functions, which are discrimination loss $L_{D}$, reconstruction loss $L_{R}$, and classification loss $L_{C}$. The discrimination loss represents the distance between the real data *T*(*x*) and the synthetic data T(G(z)) in the low-dimensional manifold space after dimensionality reduction by the transverter. The reconstruction loss represents the distance between the recovered data R(T(x)) and the original real data x obtained after the encoding and decoding processes of the transverter and the restorer. The classification loss represents the probability of whether the generated temporal data belong to a particular intention class c or not. TCGAN uses the joint training mechanism. $\theta_{G}$, $\theta_{T}$, $\theta_{R}$, and $\theta_{C}$ denote the parameters of the generator *G*, transverter *T*, restorer *R*, and classifier *C*, respectively. The joint training mechanism of TCGAN is shown in Figure 5.

The expressions of discrimination loss $L_{D}$, reconstruction loss $L_{R}$, and classification loss $L_{C}$ are shown in Equations (3)–(5).

The generator expects to generate synthetic data similar to the real data, while the transverter is responsible for transforming the real and synthetic data into the same low-dimensional manifold space and comparing the distances between them. In order to obtain synthetic data that are more similar to the real data, we need to minimize the value of the discrimination loss. The role of the transverter and the restorer is to ensure that the dimensionality reduction process is reasonable and reversible, and their training goal is to minimize the reconstruction loss. We expect the network to eventually generate temporal data that match the expected intention label, i.e., the classifier has the highest probability of classifying with the specified intention label, at which point the classification loss is minimized.

TCGAN uses the joint training mechanism, and the three loss functions are trained through the following method:

(6) $$\underset{\theta_{G},\theta_{T},\theta_{R},\theta_{C}}{\min}\left(L_{D}+\lambda L_{R}+\eta L_{C}\right)$$

Since the output of the data changes in dimensionality after both the restorer and classifier, two parameter terms λ and η (*λ*, *η* ≥ 0) are added to balance the effect of the restorer and classifier on the common part of the network. We changed the discrimination function to a distance representation instead of a true/false probability judgment, so TCGAN no longer uses the traditional GAN adversarial training model, but directly finds the minimum loss function. In practice, we can choose the appropriate values of *λ* and η according to the final generated effect. When the value of λ is small, the network focuses on downscaling the data to the manifold space with the intrinsic dimension, but some key temporal information may be lost in the downscaling process. When the value of λ is large, the network focuses on the reversibility of the dimensionality reduction process, but may not be able to transform the data into a flow space close to the intrinsic dimension, resulting in final synthetic data that differ significantly from the real data. In all experiments in Section 5, we set λ=1 and η=0.8.

This section proposes a variant model of GAN, TCGAN, which can be used to generate multiclass, multivariate temporal data for intention recognition. As mentioned in the previous section, some distance $d^{*}$ between the temporal sets is used in both the discrimination loss $L_{D}$ and reconstruction loss $L_{R}$ of TCGAN for calculation. This new distance metric for temporal point sets will be investigated in the next section.

## 4. IH-TCGAN for Generating Intention Recognition Data

The intention recognition data are multivariate time-series data. However, existing distance measures, such as Euclidean distance, Hausdorff distance [27], and Fréchet distance [28], cannot accurately quantify the similarity of time series. Among them, the Euclidean distance and Hausdorff distance do not take into account the effect of the time order in time series data, and the Fréchet distance is only applicable to the two-dimensional variable case. Next, we propose a new time-series distance metric to address this problem. In Ref. [22], the authors propose the use of Hausdorff distance as the distance between two sets for the picture generation problem. However, considering that the intention recognition data are time-ordered temporal data, in this section, we propose an improved time-regularized Hausdorff distance based on the Hausdorff distance and use it to compute the loss function.

### 4.1. Hausdorff Distance

**Figure 6:**
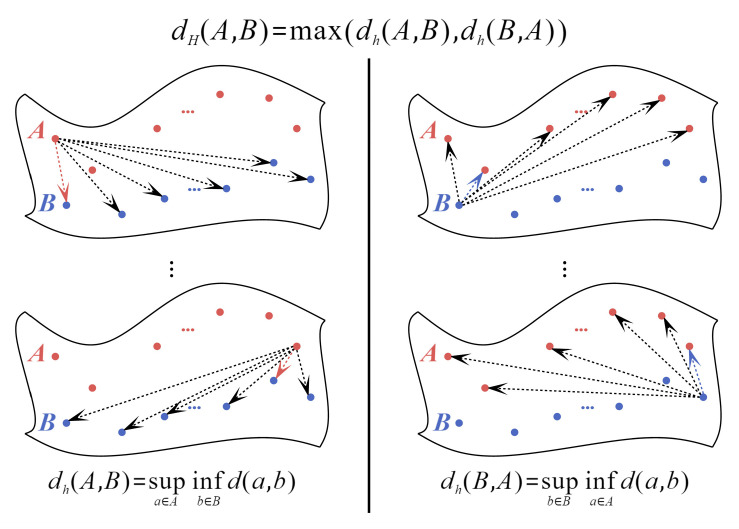
Hausdorff distance in manifold space.

The intention recognition data are multivariate time-series data, where each entry is essentially a point set with temporal order. After the transverter, its state dimension may change, but it remains consistent with the input in the temporal dimension. We need to measure distances between sets of temporal points, not between individual points. The Hausdorff distance [27] is one of the common distance measures used to calculate the distance between sets of points and generally refers to the bidirectional Hausdorff distance, which can be expressed as:

(7) $$d_{H}(A,B)=\max\left\{d_{h}(A,B),d_{h}(B,A)\right\}=\max\left\{\underset{a\in A}{\sup}\;\underset{b\in B}{\inf}\;d(a,b),\;\underset{b\in B}{\sup}\;\underset{a\in A}{\inf}\;d(a,b)\right\}$$

(8) $$d(a,b)={\left\|a-b\right\|}_{2}$$

where a and b denote points in the point sets A and B, respectively. $d_{h}(A,B)$ and $d_{h}(B,A)$ denote the unidirectional Hausdorff distance between the point sets A and B. d(a,b) denotes the Euclidean distance between points a and b. In our study, the point sets A and B can represent real data T(x) and synthetic data T(G(z)) located in the same manifold with the same dimension, or the initial input real data x and the reconstructed data R(T(x)) obtained by recovery. Figure 6 illustrates the Hausdorff distance in the manifold space.

As shown in Figure 6, the unidirectional Hausdorff distance $d_{h}(A,B)$ from the set of points A to B is the maximum of all red dashed lines, and the unidirectional Hausdorff distance $d_{h}(B,A)$ from the set of points B to *A* is the maximum of all blue dashed lines. The bidirectional Hausdorff distance $d_{H}(A,B)$ is the maximum of the two unidirectional Hausdorff distances.

### 4.2. Improved Hausdorff Distance

**Figure 7:**
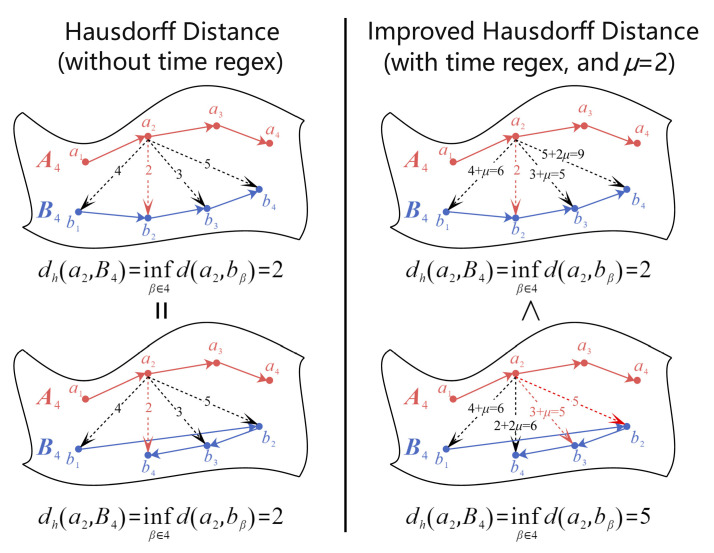
Difference between Hausdorff distance and IH distance.

Considering that the intention recognition data point sets all have temporal attributes, we assume that each sample has τ moment points, then the temporal point sets can be represented as $A_{\tau }=(a_{1},a_{2},\ldots ,a_{\tau })$ and $B_{\tau }=(b_{1},b_{2},\ldots ,b_{\tau })$. For any moment α,β∈τ, the Hausdorff distance between the set of time-series points $A_{\tau }$ and $B_{\tau }$ can be expressed as:

(9) $$d_{H}(A_{\tau },B_{\tau })=\max\left\{\underset{\alpha \in \tau }{\sup}\;\underset{\beta \in \tau }{\inf}\;d(a_{\alpha },b_{\beta }),\;\underset{\beta \in \tau }{\sup}\;\underset{\alpha \in \tau }{\inf}\;d(a_{\alpha },b_{\beta })\right\}$$

(10) $$d(a_{\alpha },b_{\beta })={\left\|a_{\alpha }-b_{\beta }\right\|}_{2}$$

where $a_{\alpha }$ denotes the point at the α-th moment in the point set $A_{\tau }$ and $b_{\beta }$ denotes the point at the β-th moment in the point set $B_{\tau }$. We can find that, although the representation of time is introduced in the above equation, the difference in time order is not actually considered. Either α=β or α≠β does not affect the final calculated Hausdorff distance. The intention recognition data are multivariate time series, and their temporal order affects the final similarity measure when comparing two pieces of data. Therefore, we need to make improvements to the common Hausdorff distance. We add a time regularization term to the above equation so that the bidirectional Hausdorff distance can represent both the difference in dimensions of the manifold space and consider the back-and-forth relationship of the time dimension. The improved Hausdorff (IH) distance formula is as follows:

(11) $$d_{IH}(A_{\tau },B_{\tau })=\max\left\{\underset{\alpha \in \tau }{\sup}\;\underset{\beta \in \tau }{\inf}\;d_{\mu }(a_{\alpha },b_{\beta }),\;\underset{\beta \in \tau }{\sup}\;\underset{\alpha \in \tau }{\inf}\;d_{\mu }(a_{\alpha },b_{\beta })\right\}$$

(12) $$d_{\mu }(a_{\alpha },b_{\beta })=d(a_{\alpha },b_{\beta })+\mu \left|\alpha -\beta \right|={\left\|a_{\alpha }-b_{\beta }\right\|}_{2}+\mu \left|\alpha -\beta \right|$$

where *μ*|*α* − *β*| is the time regularization term introduced to represent the temporal difference between different points in the two temporal point sets. μ is the parameter of the time regularization term and *μ* ≥ 0. The value of μ can be determined by performing cross-validation on the specific dataset. Figure 7 visualizes the difference between the normal Hausdorff distance and IH distance.

As shown in Figure 7, when the time order of the points in the point set $B_{\tau }$ changes, the Hausdorff distance without the time regularization term does not change, thus making it impossible to represent this change in time order. However, the IH distance with the addition of the time regularization term changes accordingly. This variation reflects well the time order relationship of the data points and fits well with our multivariate time-series dataset.

### 4.3. TCGAN with Improved Hausdorff Distance

We replace the computation of the discrimination loss and reconstruction loss in TCGAN with IH distance, which together form the final IH-TCGAN model. The loss function of IH-TCGAN is as follows:

(13) $$L_{D}=d_{IH}(T(x),T(G(z)))$$

(14) $$L_{R}=d_{IH}(x,R(T(x)))$$

(15) $$L_{C}=-E\left[\log P(C=c|T(\circ ))\right]$$

where $d_{IH}$ is the IH distance between two sets of time-series points. The meaning of the remaining letters is the same as before.

We use the IH distance to calculate the distance between real and synthetic data, and real and reconstructed data. Using the IH distance has the following advantages.

1. From Equations (14) and (15), it can be seen that the IH distance consists of two parts: the spatial distance and the temporal distance in the manifold space. The larger the IH distance between two time-series point sets, the larger the spatial difference and the temporal difference. Similarly, if the IH distance is smaller, then it means that the difference between the two in space and time is smaller and the two are closer. The IH distance is non-negative. The value can be taken close to the minimum value of 0 when and only when both spatial and temporal properties of the two temporal point sets are almost identical.
2. The IH distance traverses each mapped data and uses the maximum–minimum distance to minimize the differences between the datasets. The discrimination loss and reconstruction loss are calculated by the IH distance. As the discrimination loss decreases, the distribution of the synthetic data will slowly approach the distribution of the real data, and eventually the generator can generate synthetic data similar to the real data. As the reconstruction loss decreases, the difference between the real data and the reconstructed data becomes smaller, which means that the real data can still be restored to the original input data as much as possible. At this point, the transverter achieves the dimensionality reduction function without losing too much key feature information, which ensures the rationality of the transformer dimensionality reduction process.

IH-TCGAN still uses the joint training mechanism, and the three loss functions are trained by Equation (6).

We take λ=1 and η=0.8.

## 5. Experimental Analysis

To validate the generative effect of our proposed IH-TCGAN model, in this section we conducted experiments and analysis with two datasets (human activity recognition dataset and target intention recognition dataset). These experiments focused on the following questions:

- Can IH-TCGAN generate multiclass, multivariate time-series data?
- Does our proposed IH distance considering time order have more advantages than other distance methods?
- Can the generated intention recognition data be applied to an intention recognition model and obtain a more accurate recognition result?

### 5.1. Experimental Data and Environment

The main dataset used for the experiments was the target intention recognition dataset [16]. The experimental data were provided by the simulation system. Time series information of the air target while executing different actions with different intentions was obtained by the system backend. The dataset had 6 classes, which are attack, reconnaissance, surveillance, cover, interference, and retreat. Each piece of data in the dataset included 12 time-varying target characteristics, specifically height, velocity, acceleration, heading angle, azimuth, etc. The sampling step for each sample in the dataset was 10, and the sampling interval was 3 s.

In addition, to further validate the generation effect of IH-TCGAN, the datasets used in other studies on multivariate temporal data enhancement methods were referenced. We chose the human activity recognition dataset [29], which is widely used in the field of temporal data generation, as the second dataset for our experiments. The human activity recognition dataset is similar to the target intention recognition dataset in that it is also a time-series dataset with multiple classes and multiple variables. Time series information is collected from sensors placed on the chest and ankles while the user is performing different activities. The dataset has seven classes, which are bending1, bending2, cycling, flying, sitting, standing, and walking. Each piece of data in the dataset includes six time-varying features. The sampling frequency for each activity is 20 Hz, the clock is 250 milliseconds, and the total duration is 120 s.

The experimental computer system was Windows 10, and Python version was 3.8.0. NVIDIA GeForce RTX 3060 GPU and CUDA 11.0 were used for acceleration, and the PyTorch 1.8.0 deep learning framework was used.

### 5.2. Benchmarks and Evaluation Metrics

We chose two groups of benchmark methods to demonstrate the effectiveness of IH-TCGAN. The first group was based on GAN methods, specifically TimeGAN [2], RCGAN [5], PART-GAN [6], LSGAN [30], and CWGAN [31]. These methods can provide help on the multivariate time-series generation problem, and we hoped to demonstrate that our proposed IH-TCGAN can generate better quality time-series data through the first group of comparison experiments. The second group was the methods using other distance methods as loss functions, specifically H-TCGAN, L2-TCGAN, and F-TCGAN. H-TCGAN, L2-TCGAN, and F-TCGAN denote the use of ordinary Hausdorff distance, L2-norm (Euclidean norm), and F-norm (Frobenius norm), instead of our proposed IH distance, as the loss function of the TCGAN model, respectively. The purpose of the second group of comparison was to verify that our proposed IH distance method had greater advantages.

We used quantitative and qualitative methods to compare the effects of the above benchmark models. The quantitative evaluation metrics included Discriminative Score, Predictive Score, Precision, Recall, and F1 Score. The qualitative evaluation focused on visualization to demonstrate visually whether the model can generate multiclass, multivariate time-series data.

- Discriminative Score, Precision, Recall, and F1 Score. Discriminative Score is derived from TimeGAN [2]. Based on the TSTR (train-on-synthetic and test-on-real) methodology, a 2-layer LSTM time-series classification model was trained using synthetic data to distinguish between the real data series and synthetic data series. The respective error values were calculated as the Discriminative Score. Further, the Precision, Recall, and F1 score were calculated based on this classification model. It is important to notice that the smaller the value of the Discriminative Score, the better the model performance. In contrast, the larger the Precision, Recall and F1 Score are, the better.
- Predictive Score. Predictive Score was also derived from TimeGAN [2]. A 2-layer LSTM sequence prediction model was trained using synthetic data to predict the multivariate time vector in the next step based on the previous step. Prediction performance is measured using mean absolute error (MAE), which is Predictive Score. Predictive Score can test whether the model is able to capture the time-varying temporal dynamics and conditional distribution over time. The smaller the value of the Predictive Score, the better the prediction performance of the model.
- Visualization. We used the t-SNE method to reduce the dimensionality of the synthetic data and plot it in the two-dimensional image. This provides a visual representation of the distribution of the various classes in the synthetic data. We used different colors to indicate different classes to verify whether the model can generate time series data with distinctive features for multiple classes.

### 5.3. Results and Analysis

#### 5.3.1. Comparative Analysis of GAN-Based Methods

For the purpose of fair comparison, a standard Gaussian distribution of (0,1) was used for the original noise distribution of all models. The optimal values of the network parameters of the GAN-based methods were obtained through several comparison experiments. The parameters of TimeGAN, RCGAN, PART-GAN, LSGAN, CWGAN, and IH-TCGAN are shown in Table 1.

We generated new data using the above method on both datasets and calculated Discriminative Score, Predictive Score, Precision, Recall, and F1 Score. The experimental results of the GAN-based methods are shown in Table 2 and Table 3. The smaller the Discriminative Score and Predictive Score the better, and the larger the remaining metrics the better.

Table 2 and Table 3 list the performance metrics of the six GAN-based methods on the target intention recognition dataset and the human activity recognition dataset. As shown in Table 2, our proposed IH-TCGAN has optimal performance on all five quantitative metrics when compared with other GAN-based methods. The next best performing methods are PART-GAN and CWGAN. In addition, we found that the results of various methods on the human activity recognition dataset are generally better than those on the target intention recognition dataset. This may be due to the fact that the sample data in the human activity recognition dataset have better temporal characteristics, and the temporal features differ more significantly among different classes.

#### 5.3.2. Comparative Analysis of Different Distance Methods

We changed the loss function calculation in TCGAN and used different distance methods as the loss function. The experimental results are shown in Table 4 and Table 5. The parameters of each method are the same as Table 1, except the distance formula in the loss function is different.

As shown in Table 4 and Table 5, the IH-TCGAN method with our proposed IH distance has the optimal performance. This indicates that the time regularization term we added to the Hausdorff distance plays an important role. The experimental result of H-TCGAN is suboptimal, which indicates that Hausdorff distance has a general advantage in the generation of sequential data. The poor performance of L2-TCGAN and F-TCGAN indicates that L2-norm and F-norm are not applicable to the problem of generating temporal feature data.

#### 5.3.3. Visualization Results

**Figure 8:**
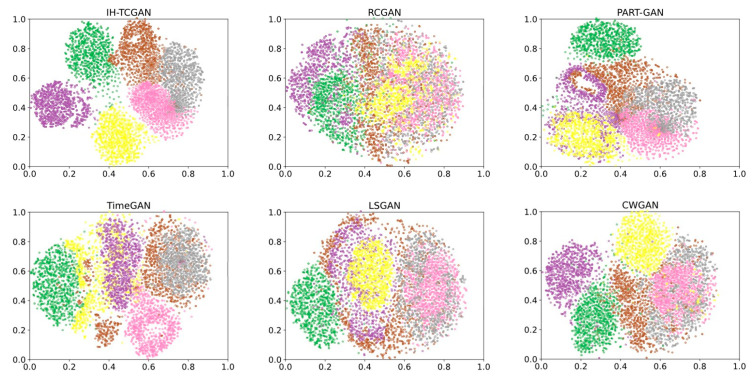
Visualization results of the GAN-based methods. The first figure shows the visualization results of our proposed IH-TCGAN.

**Figure 9:**
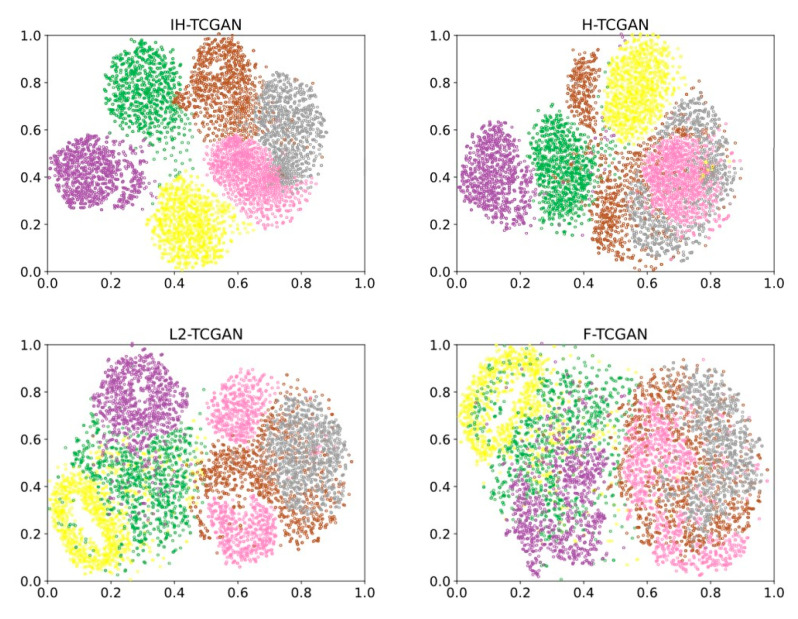
Visualization results of different distance methods. The first figure shows the visualization results of our proposed IH-TCGAN.

The visualization results can visualize the distribution of each class in the synthesized data. The t-SNE plots of the synthetic data generated by GAN-based methods and different distance methods on the target intention recognition dataset are shown in Figure 8 and Figure 9. The different colored point sets in Figure 8 and Figure 9 indicate different intention class data. The yellow point set indicates attack intention, the brown point set indicates reconnaissance intention, the pink point set indicates surveillance intention, the green point set indicates cover intention, the gray point set indicates interference intention, and the purple point set indicates retreat intention.

We find from Figure 8 and Figure 9 that the class distribution of the synthetic data generated by IH-TCGAN is better than that of the GAN-based methods and the different distance methods. In the t-SNE plot of IH-TCGAN, the same color points representing the same intention are more closely clustered, and the different sets of color points representing different intentions are distributed at a greater distance from each other. This indicates that the dimensionality reduction process of the transformer in the IH-TCGAN method successfully learns the hidden temporal characteristics in the real data, which makes the generated synthetic data with different intentions more distinctive. IH-TCGAN is capable of generating multiclass, multivariate time-series data. The t-SNE plots using other methods often show a mixture of point sets with multiple colors. This indicates that the other methods cannot learn the temporal characteristics of different classes of data correctly, which makes the class characteristics of the generated data not obvious and cannot meet the demands of multiclass generation. In addition, we also find that the brown, pink, and gray point sets representing reconnaissance, surveillance, and interference are close to each other and difficult to distinguish in multiple t-SNE plots. This indicates that the temporal characteristics of these three elements of intention data are more similar, which makes the generated synthetic data also have high similarity.

#### 5.3.4. Analysis of Exploiting Experiment

We hope that the intention recognition data generated by the IH-TCGAN model can be applied to a general intention recognition model and obtain more accurate recognition results. Therefore, we use the STABC-IR model [16] for validation. STABC-IR is an air-target intention recognition method based on bidirectional-gated recurrent unit and conditional random field with a space–time attention mechanism. In the exploiting experiments, the experiments are designed to address the following two main problems.

1. Data Scarcity Problem. In practice, the amount of real intention recognition data that can be obtained may be extremely small due to the secrecy of military data and the complex adversarial nature of the battlefield. This situation cannot provide sufficient training set samples for deep learning-based intention recognition models, resulting in inadequate training of recognition models and low recognition accuracy. Therefore, we first explore whether the intention recognition data generated by IH-TCGAN and other data generation methods can be used as a supplement to the real sample training set, and then train the recognition model to achieve better or similar recognition accuracy. In addition, this can also verify that the IH-TCGAN method can generate high-quality multiclass multivariate temporal data for intention recognition.
2. Sample Imbalance Problem. There may be differences in the number of samples for different intention labels in the intention recognition dataset. The large difference will lead to a large bias in the accuracy of the trained recognition model for classifying various intentions. The recognition models are more likely to favor the intention classes with large sample sizes and ignore the intention classes with small sample sizes. To prevent recognition models from learning a priori information with sample class proportions and being able to essentially identify different intention classes, we utilize IH-TCGAN and other data generation methods to generate more samples. They are added to the training set, thus balancing the sample classes and improving the recognition accuracy of the recognition model.

**Figure 10:**
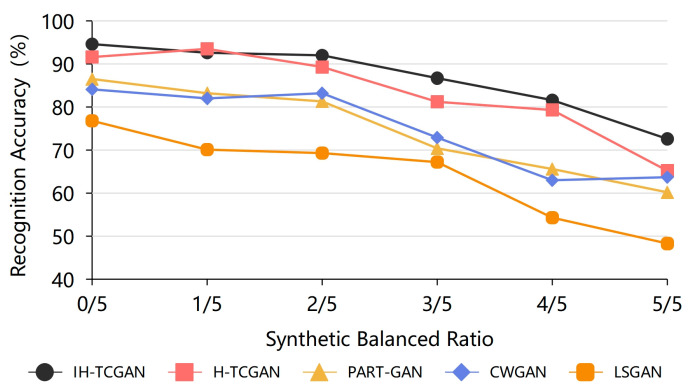
Identification results of different synthetic balanced ratios.

To address the problem of data scarcity, we design the synthetic balanced ratio to set the training set of the deep learning recognition model. The synthetic balanced ratio refers to the ratio of synthetic data samples generated by IH-TCGAN or other data generation methods to the total number of samples in the training set. Multiple training sets with the same total number of samples but a different synthetic balanced ratio are used to train the STABC-IR recognition model, where the number of samples for each intention class is the same. Based on the TSTR theory, the recognition model is tested using a test set consisting of the same real data. The intention recognition accuracy of the STABC-IR model under different synthetic balanced ratio conditions is shown in Figure 10. In addition to the IH-TAGAN method, we also selected the H-TCGAN, PART-GAN, CWGAN, and LSGAN methods with better generation effects for comparison.

As shown in Table 4, the accuracy of the intention recognition model gradually decreases with the increase in the synthetic balanced ratio. When the synthetic balanced ratio is small, the real data in the training set are greater than the synthetic data, and the recognition accuracy is higher. When the synthetic balanced ratio is larger, there are more synthetic data in the training set, which leads to the decrease in the final recognition accuracy. However, when the synthetic balanced ratio is less than 0.5 in this experiment, the decreasing effect of recognition accuracy is not obvious and can be maintained at more than 92%. This indicates that the synthetic data generated by IH-TCGAN can be applied to the training process of STABC-IR intention recognition model and obtain a more accurate recognition result. When the synthetic balanced ratio is 5/5, i.e., the training set is all synthetic data, the recognition accuracy of the data generated by the IH-TCGAN-based method is 72.6% at this time, which is still greater than the 50% accuracy rate. This indicates that intention recognition data can be generated by IH-TCGAN to solve the key problem of scarcity of real battlefield data in the field of intention recognition. In addition, it can be found that the recognition accuracy of data generated based on other methods is generally lower than that of data generated based on the IH-TCGAN method, which also reflects that the IH-TCGAN method can generate multiclass, multivariate intention recognition data of a higher quality.

**Figure 11:**
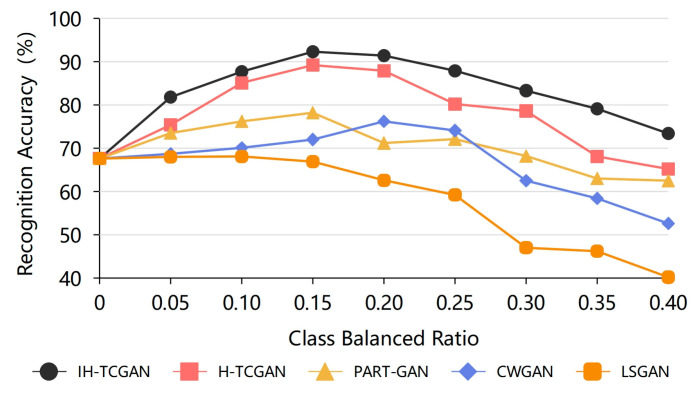
Identification results for different class balanced ratios.

To address the problem of sample imbalance, we design the class balanced ratio to set the training set of the deep learning recognition model. The class balanced ratio refers to the ratio of the number of minority intention samples to the total number of samples in the training set. Based on the class composition of the training dataset, we found that the number of samples whose intention is surveillance is very small, so data augmentation is performed for the samples with surveillance intention. More samples with the class of surveillance are generated and added to the training set using IH-TCGAN and other data generation methods. The class balanced ratio here refers to the ratio of the number of samples for surveillance intention to the total number of samples in the training set, and the number of samples for the remaining five intention classes, except for surveillance intention, is the same. In the test set, the number of samples corresponding to each intention is the same. The intention recognition accuracy of STABC-IR model under different class balanced ratio conditions is shown in Figure 11. In addition to the IH-TAGAN method, the H-TCGAN, PART-GAN, CWGAN, and LSGAN methods, which have better generation effects, are still selected for comparison.

As can be seen from Figure 11, the recognition accuracy of the STABC-IR model is low when the class balanced ratio of the surveillance intention samples is low or high. When the class balanced ratio is 0, there are no monitoring intention samples in the training set, and the recognition accuracy of the recognition model is very low, only 67.6%. When the class balanced ratio is about 0.15, the number of samples of surveillance intention in the training set is basically the same as the number of samples of each other intention, and the recognition model can obtain the highest recognition accuracy. When the class balanced ratio continues to increase, the number of surveillance intention samples in the training set at this time gradually exceeds the number of samples for each of the other intention classes. However, the recognition accuracy of the recognition model based on the data generated by the IH-TCGAN and H-TCGAN methods decreases but at a slower rate because there are still enough samples of each intention class in the training set. The recognition accuracy of recognition models based on data generated by several other generation methods decreases faster as the class balanced ratio increases. This may be due to the fact that the training set contains more synthetic data, but these other generation methods are less effective and generate synthetic data of lower quality, resulting in a rapid decrease in recognition accuracy.

Similar to the experimental results with different synthetic balanced ratios, the recognition accuracy of the data generated based on other methods is generally lower than that of the data generated based on the IH-TCGAN method, which can also reflect that the IH-TCGAN method can generate better quality temporal data for intention recognition. The recognition results with different class balanced ratios show that the IH-TCGAN method can generate samples for a few classes in the training set for intention recognition. The best recognition results can be obtained by adding the generated samples to the training set and training the recognition model on the balanced training set. This effectively solves the problems such as difficulties in training recognition models due to sample imbalance.

## 6. Conclusions

In this paper, we propose a new IH-TCGAN method for generating multiclass temporal data for intention recognition. First, we design a time-series conditional generative adversarial network for generating multiclass time-series data. The discriminator in the traditional GAN is improved into a transverter, and the restorer and classifier are added to the network structure to ensure the reversibility of the transformation process and the diversity of the synthesized samples. Second, we propose the improved Hausdorff distance considering the time order for the characteristics of time series data and use it as the loss function of TCGAN. The improved Hausdorff distance formula contains a time regularization term that characterizes the difference in time order in the time series data and can better measure the distance between two time-series sets. Finally, comparison experiments are conducted on two time-series datasets. IH-TCGAN can generate multiclass temporal data similar to real data and has better performance than other temporal data generation methods and different distance measurement methods. The exploiting experiment shows that the intention recognition temporal data generated by IH-TCGAN can be used to train existing recognition models with good results.

The IH-TCGAN method can greatly improve the problem of sparse and unbalanced real-measurement datasets in intention recognition. Moreover, IH-TCGAN can also be used in other broader scenarios of multiclass, multivariate time-series data generation. In the future, we plan to conduct research on the possible imperfections and incompleteness of the dataset to further improve the application of IH-TCGAN.

## Figures and Tables

**Table 1:** Parameters of the GAN-based methods.

Methods	Parameters	Value
TimeGAN/RCGAN/PART-GAN/LSGAN/CWGAN	Learning rate	0.0001
	Epoch	2000
	Batch size	256
	Hidden layer	LSTM layer 128, 64, 64
IH-TCGAN (We proposed)	λ	1
	η	0.8
	μ	1.2
	Learning rate	0.0001
	Epoch	2000
	Batch size	256
	Hidden layer	LSTM layer 128, 64, 64

**Table 2:** Experimental results of GAN-based methods on the target intention recognition dataset.

Methods	Target Intention Recognition Dataset				
	Discriminative Score	Predictive Score	Precision	Recall	F1 Score
IH-TCGAN	0.451	0.297	0.934	0.912	0.9229
TimeGAN	0.597	0.463	0.911	0.819	0.8625
RCGAN	0.712	0.590	0.823	0.814	0.8185
PART-GAN	0.484	0.350	0.897	0.892	0.8945
LSGAN	0.584	0.504	0.831	0.801	0.8158
CWGAN	0.517	0.368	0.859	0.843	0.8509

**Table 3:** Experimental results of GAN-based methods on the human activity recognition dataset.

Methods	Human Activity Recognition Dataset				
	Discriminative Score	Predictive Score	Precision	Recall	F1 Score
IH-TCGAN	0.243	0.126	0.971	0.930	0.9501
TimeGAN	0.387	0.314	0.940	0.827	0.8799
RCGAN	0.590	0.498	0.843	0.770	0.8048
PART-GAN	0.307	0.191	0.951	0.901	0.9253
LSGAN	0.412	0.446	0.873	0.819	0.8451
CWGAN	0.356	0.247	0.907	0.876	0.8912

**Table 4:** Experimental results of different distance methods on the target intention recognition dataset.

Methods	Target Intention Recognition Dataset				
	Discriminative Score	Predictive Score	Precision	Recall	F1 Score
IH-TCGAN	0.451	0.297	0.934	0.912	0.9229
H-TCGAN	0.492	0.358	0.841	0.875	0.8577
L2-TCGAN	0.641	0.346	0.865	0.803	0.8328
F-TCGAN	0.713	0.501	0.806	0.767	0.7860

**Table 5:** Experimental results of different distance methods on the human activity recognition dataset.

Methods	Human Activity Recognition Dataset				
	Discriminative Score	Predictive Score	Precision	Recall	F1 Score
IH-TCGAN	0.243	0.126	0.971	0.930	0.9501
H-TCGAN	0.278	0.267	0.932	0.843	0.8853
L2-TCGAN	0.324	0.284	0.847	0.879	0.8627
F-TCGAN	0.474	0.417	0.834	0.810	0.8218

## Data Availability

The data presented in this study are available on request from the corresponding author. Some of the data can be found at https://www.payititi.com/opendatasets/show-25928.html (accessed on 20 March 2023).

## References

[1] Goodfellow I.J., Pouget-Abadie J., Mirza M., Xu B., Warde-Farley D., Ozair S., Courville A., Bengio Y. (2020). Generative adversarial networks. Commun. ACM.

[2] Yoon J., Jarrett D., Van der Schaar M. Time-series generative adversarial networks. Proceedings of the Advances in Neural Information Processing Systems.

[3] Asre S., Anwar A. (2022). Synthetic Energy Data Generation Using Time Variant Generative Adversarial Network. Electronics.

[4] Snow D. (2020). MTSS-GAN: Multivariate Time Series Simulation Generative Adversarial Networks. https://ssrn.com/abstract=3616557.

[5] Esteban C., Hyland S.L., Rätsch G. (2017). Real-valued (medical) time series generation with recurrent conditional gans. arXiv.

[6] Wang S., Rudolph C., Nepal S., Grobler M., Chen S. (2020). PART-GAN: Privacy-preserving time-series sharing. Artificial Neural Networks and Machine Learning–ICANN 2020: 29th International Conference on Artificial Neural Networks, Bratislava, Slovakia, 15–18 September 2020, Proceedings, Part I 29.

[7] Paul J., Michael B.-S., Pedro M., Shubham K., Rajbir S.N., Valentin F., Jan G., Tim J. (2021). PSA-GAN: Progressive Self Attention GANs for Synthetic Time Series. arXiv.

[8] Remlinger C., Mikael J., Elie R. (2022). Conditional loss and deep euler scheme for time series generation. Proc. AAAI Conf. Artif. Intell..

[9] Li D., Chen D., Jin B., Shi L., Goh J., Ng S.-K. (2019). MAD-GAN: Multivariate anomaly detection for time series data with generative adversarial networks. Artificial Neural Networks and Machine Learning–ICANN 2019: Text and Time Series: 28th International Conference on Artificial Neural Networks, Munich, Germany, 17–19September 2019, Proceedings, Part IV.

[10] Du B., Sun X., Ye J., Cheng K., Wang J., Sun L. (2021). GAN-based anomaly detection for multivariate time series using polluted training set. IEEE Transactions on Knowledge and Data Engineering.

[11] Lin Z., Jain A., Wang C., Fanti G., Sekar V. Using gans for sharing networked time series data: Challenges, initial promise, and open questions. Proceedings of the ACM Internet Measurement Conference.

[12] Desai A., Freeman C., Wang Z., Beaver I. (2021). TimeVAE: A variational auto-encoder for multivariate time series generation. arXiv.

[13] Fu R., Chen J., Zeng S., Zhuang Y., Sudjianto A. (2020). Time Series Simulation by Conditional Generative Adversarial Net. Int. J. Mech. Ind. Eng..

[14] Chen Y., Kempton D.J., Ahmadzadeh A., Angryk R.A. (2021). Towards synthetic multivariate time series generation for flare forecasting. Artificial Intelligence and Soft Computing: 20th International Conference, ICAISC 2021, Virtual Event, 21–23 June 2021, Proceedings, Part I 20.

[15] Huang X., Li Q., Tai Y., Chen Z., Liu J., Shi J., Liu W. (2022). Time series forecasting for hourly photovoltaic power using conditional generative adversarial network and Bi-LSTM. Energy.

[16] Wang S., Wang G., Fu Q., Song Y., Liu J., He S. (2023). STABC-IR: An air target intention recognition method based on bidirectional gated recurrent unit and conditional random field with space-time attention mechanism. Chin. J. Aeronaut..

[17] Mirza M., Osindero S. (2014). Conditional generative adversarial nets. arXiv.

[18] Yu H., Chen X., Li Z., Zhang G., Liu P., Yang J., Yang Y. (2019). Taxi-Based Mobility Demand Formulation and Prediction Using Conditional Generative Adversarial Network-Driven Learning Approaches. IEEE Trans. Intell. Transp. Syst..

[19] Xue Y., Tong W., Neri F., Zhang Y. (2022). PEGANs: Phased Evolutionary Generative Adversarial Networks with Self-Attention Module. Mathematics.

[20] Xue Y., Chen C., Słowik A. (2023). Neural Architecture Search Based on A Multi-objective Evolutionary Algorithm with Probability Stack. IEEE Transactions on Evolutionary Computation.

[21] Xue Y., Qin J. (2022). Partial Connection Based on Channel Attention for Differentiable Neural Architecture Search. IEEE Trans. Ind. Inform..

[22] Li W., Liang Z., Ma P., Wang R., Cui X., Chen P. (2021). *Hausdorff* GAN: Improving GAN Generation Quality with *Hausdorff* Metric. IEEE Trans. Cybern..

[23] Tenenbaum J.B., de Silva V., Langford J.C. (2000). A Global Geometric Framework for Nonlinear Dimensionality Reduction. Science.

[24] Campadelli P., Casiraghi E., Ceruti C., Rozza A. (2015). Intrinsic Dimension Estimation: Relevant Techniques and a Benchmark Framework. Math. Probl. Eng..

[25] Goodfellow I., Bengio Y., Courville A. (2016). Deep Learning.

[26] Levina E., Bickel P. (2004). Maximum likelihood estimation of intrinsic dimension. Advances in Neural Information Processing Systems.

[27] Atallah M.J. (1983). A linear time algorithm for the Hausdorff distance between convex polygons. Inf. Process. Lett..

[28] Alt H., Godau M. (1995). Computing the Fréchet distance between two polygonal curves. Int. J. Comput. Geom. Appl..

[29] Palumbo F., Gallicchio C., Pucci R., Micheli A. (2016). Human activity recognition using multisensor data fusion based on Reservoir Computing. J. Ambient. Intell. Smart Environ..

[30] Mao X., Li Q., Xie H., Lau R.Y.K., Wang Z., Smolley S.P. Least squares generative adversarial network. Proceedings of the IEEE International Conference on Computer Vision.

[31] Zhang Y., Sun B., Xiao Y., Xiao R., Wei Y. (2019). Feature augmentation for imbalanced classification with conditional mixture WGANs. Signal Process. Image Commun..

